# Redox Response in Postoperative Metabolic and Bariatric Surgery: New Insights into Cardiovascular Risk Markers

**DOI:** 10.3390/nu17243821

**Published:** 2025-12-06

**Authors:** Ruanda Pereira Maia, Sandra Fernandes Arruda, Ariene Silva do Carmo, Patrícia Borges Botelho, Kênia Mara Baiocchi de Carvalho

**Affiliations:** 1 Graduate Program in Human Nutrition, Department of Nutrition, Faculty of Health Science, University of Brasília, Brasília 70910-900, Brazil; ruanda_maia@yahoo.com.br (R.P.M.); arienecarmo@gmail.com (A.S.d.C.); kenia@unb.br (K.M.B.d.C.); 2 Department of Clinical and Social Nutrition, Nutrition School, Federal University of Ouro Preto (UFOP), Ouro Preto 35400-000, Brazil; 3 Faculty of Applied Sciences, State University of Campinas, Campinas 13083-970, Brazil; patriciaborges.nutri@gmail.com

**Keywords:** obesity, metabolic and bariatric surgery, oxidative stress, redox, cardiovascular risk

## Abstract

**Background/Objectives:** Metabolic and bariatric surgery (MBS) promotes improved redox response and weight loss and reduced cardiovascular risk. However, there is still no consensus on whether some of these results may be affected years after the surgery. This study evaluated the association between redox response and cardiovascular risk markers following MBS. **Methods:** Patients (*n* = 91) of both sexes who underwent MBS 2–7 years ago were evaluated. Antioxidant enzymatic activity (catalase, superoxide dismutase, glutathione-S-transferase, and glutathione peroxidase) and oxidative damage (malondialdehyde and carbonylated protein) were quantified. Blood pressure, glucose, insulin, triglyceride/glucose (TyG) index, LDL-C, HDL-C, non-HDL-C, triglyceride (TG), and cholesterol were analyzed. Principal component analysis (PCA) and generalized linear models were used. **Results:** The participants had a mean age of 39.82 ± 7.87 years, and a current body mass index of 29.53 ± 5.01 kg/m^2^. The PCA identified two patterns: enzymatic antioxidant activity (PC1) and oxidative damage (PC2). No association was found between PC1 and cardiovascular risk markers. A positive association was observed between PC2 and diastolic blood pressure (β: 6.79, 95% confidence intervals [CI]: 1.97; 11.61), TyG index (β: 0.13, 95% CI: 0.05; 0.21), total cholesterol (β: 15.17, 95% CI: 3.61; 26.72), TG (β: 25.88, 95% CI: 8.58; 43.18; *p* = 0.003), and non-HDL-C (β: 10.91, 95% CI: 0.02; 21.88). **Conclusions:** Oxidative damage markers were positively associated with diastolic blood pressure, TyG index, TG, total cholesterol, and non-HDL-C levels after MBS. However, further studies are required to confirm and elucidate these findings.

## 1. Introduction

Obesity increases the production of reactive oxygen species (ROS) and reduces antioxidant defenses, thereby disrupting redox homeostasis [1]. Impairment of redox homeostasis in obesity has been associated with the onset of insulin resistance and metabolic diseases, such as hypertension, dyslipidemia, and metabolic syndrome, increasing cardiovascular risk [2,3]. However, a reduction in excess body fat improved redox and inflammatory homeostasis [4].

Metabolic and bariatric surgery (MBS) is considered the most effective intervention for sustained weight loss in individuals with obesity, more so than clinical treatments [5,6]. Pinto et al. [7] observed a reduction in oxidative damage to lipids, in superoxide dismutase, and in the triglyceride/glucose index (TyG), and an improvement in the lipid profile and other cardiometabolic risk markers at 3 and 12 months post-surgery compared with those at baseline. Similarly, a reduction in oxidative damage to lipids was reported after 12 months of MBS [8]. However, after 2 years, an increase in thiobarbituric acid reactive substances (TBARSs) and a decrease in the antioxidant response were observed. According to the authors, the imbalance between pro-oxidants and antioxidants may be partly attributable to weight regain (WR) and increased calorie consumption [8]. Additionally, in another follow-up study, although a decrease in TBARS was observed 24 months after MBS, the TBARS levels returned to baseline values 72 months after surgery. This late response was partially explained by WR and insufficient supplement use [9].

Although MBS is recognized for an improvement in oxidative stress [4], effectiveness in weight loss, and reduction in major adverse cardiovascular events [10], weight regain may also occur in some individuals [11]. In a systematic review, it was identified that at least 1 in 6 patients after MBS had weight regain ≥ 10% [12]. Over the years of MBS, due to adaptive processes or discontinuation of clinical monitoring, it is common for patients to return to an unhealthy lifestyle [13]. Courcoulas et al. [14] highlighted the need for lifelong monitoring to help reduce the risk of nutritional deficiencies, weight regain, and alcohol use disorders, among others. Weight regain can be harmful due to the worsening redox response, increased possibility of comorbidity recurrence [15], and deterioration in quality of life [16]. Therefore, long-term clinical monitoring is important for maintaining these results.

Although MBS promotes greater loss of excess body fat and has been associated with improved redox and inflammatory homeostasis, there is no consensus on whether this change is sustained years after surgery. The hypothesis is that individuals after MBS with a poorer redox response may have a higher cardiovascular risk. Thus, this study investigated the association between the redox response pattern and cardiovascular risk markers 2–7 years after MBS.

## 2. Materials and Methods

### 2.1. Participants

This was an observational study performed with a single assessment (the baseline data) of the Randomized Clinical Trial entitled “Nutrition and Online Resistance Exercise—NERON Study.” This study evaluated clinical and metabolic outcomes in adults (18–60 years old), of both sexes, who underwent Roux-en-Y Gastric Bypass or Laparoscopic Sleeve Gastrectomy between 2 and 7 years ago. This period was defined as the probability of maintaining weight loss [17] or evolving toward WR [18]. The exclusion criteria were the same as those for the clinical trial: physically active individuals; pregnant women; patients with decompensated chronic diseases, pacemakers or psychiatric disorders.

The sample size calculation considered type I errors of 5% and power of 80% for the evaluation of independent variables through principal component analyses and linear regression models, which resulted in a sample size of 70 participants [19].

**Figure 1:**
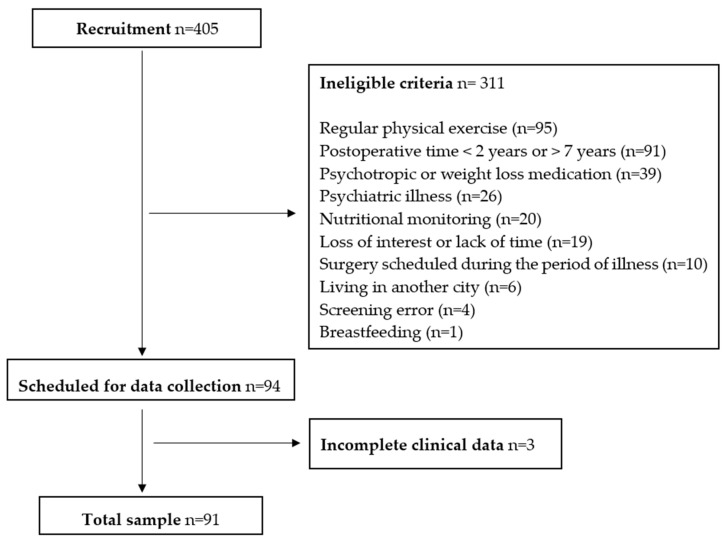
Flowchart of baseline from NERON study participants throughout the recruitment and sample selection period.

Overall, 405 individuals were recruited for this study, and 311 were excluded according to ineligibility criteria. Following confirmation of preselection data, 94 participants’ data were collected and 91 were included in this study. This resulted in an average enrolment rate of 22.47% (Figure 1).

### 2.2. Sociodemographic Data

An online semi-structured questionnaire was used to collect sociodemographic variables, surgical data, use of supplements, and smoking status. The Alcohol Use Disorders Identification Test [20] was used to characterize alcohol use by patients.

### 2.3. Clinical Measurements

Weight and body composition were measured using the equipment InBody^®^ model 720 (Biospace, Seoul, Republic of Korea) with a capacity of 250 kg and precision of 100 g. Weight and height were measured, and body mass index (BMI) was calculated using the recorded weight and squared height and classified according to the World Health Organization criteria [21].

The ideal weight of the patients was calculated using a BMI value of 25 kg/m^2^ as a reference. Excess weight was calculated as the difference between preoperative weight (kg) and ideal weight (kg). The percentage of excess weight loss was calculated as follows: ([preoperative weight—current weight]/excess weight) × 100. Total weight loss (%) was calculated using the following formula: ([current weight—preoperative weight]/current weight) × 100. Moreover, the percentage of WR was considered significant when >10% [15].

Blood pressure values were measured according to the recommendations of the American Heart Association [22] using an oscillometric device (Omron^®^ 7051T; Kyoto, Japan) validated for adults and previously calibrated. Systolic and diastolic blood pressures were measured thrice with a 60 s interval between each measurement. The final result was considered the average of the arm in which two measurements were noted.

### 2.4. Dietary Assessment

Dietary intake was collected through two 24 h dietary recalls applied on nonconsecutive, random days; the first was conducted in person and the second, over telephone. The five-step multiple-pass method from the United States Department of Agriculture was used for standardization in data collection [23]. Data were analyzed using the Brazil-Nutri software [24], and the multiple source method was used to estimate dietary usual intake [25]. The total antioxidant capacity of the diet (dTAC) was estimated using TAC values of foods, beverages, and supplements as described by Carlsen et al. [26]. The table contains the TAC values expressed as mmol Fe/100 g of >3100 foods and preparations determined by ferric-reducing assay. Therefore, the dTAC is a sum of all TAC values of each food item consumed, in mmol/1000 kcal. For foods whose TAC values were unavailable, data of foods that are botanically similar were used.

### 2.5. Biochemical Analyses

#### 2.5.1. Biological Sample

After 12 h of fasting, blood samples were collected from each participant by a private partner laboratory accredited by the American College of Pathology. The partner laboratory performed the following analyses: blood glucose, basal insulin, HbA1c, total cholesterol, triglyceride (TG), high-density lipoprotein cholesterol (HDL-C), low-density lipoprotein cholesterol (LDL-C), and C-reactive protein (CRP). Non-high-density lipoprotein cholesterol (Non-HDL-C) levels were estimated based on the difference between total and HDL cholesterol levels. Homeostatic Model Assessment was performed using the formula: ([fasting insulin {U/mL}] × [fasting glucose {mmol/L}])/22.5 [27]. Furthermore, the TyG was calculated from TG and fasting glycemia using the formula (ln [fasting TGs {mg/dL} × fasting glycemia {mg/dL}])/2 [28].

#### 2.5.2. Redox Response Biomarkers

The activity of antioxidant enzymes (catalase, superoxide dismutase, glutathione-S-transferase, and glutathione peroxidase) and oxidative damage to lipids (malondialdehyde [MDA]) and proteins (carbonylated protein) were quantified in the red blood cells, serum, and plasma, respectively (Table 1). All analyses were performed in triplicate.

##### Sample Homogenate

The red blood cells were lysed in a 1:1 proportion (500 μL red blood cell and 500 μL deionized water) and resuspended in a microtube. The activities of catalase (CAT), glutathione peroxidase (GPx), glutathione reductase, and glutathione-S transferase were determined in the same homogenate (50 μL of red blood cell lysate, 940 μL 50 mmol/L potassium phosphate buffer [pH 7.2] with ethylenediaminetetraacetic acid (EDTA) 0.5 mmol/L, and 10 μL 1 mmol/L phenylmethylsulfonyl fluoride (PMSF) with ethanol). For the superoxide dismutase (SOD) assay, 10 μL of red blood cell lysate was used, 980 μL 50 mmol/L potassium phosphate buffer (pH 7.8) with EDTA 0.5 mmol/L and 10 μL 1 mmol/L PMSF with ethanol.

Total protein concentration in red blood cells homogenate and plasma was measured using the method described and modified by Lowry et al. and Hartree, respectively [29].

##### CAT Assay

The CAT activity was evaluated according to the method described by Joanisse and Storey [30]. Specifically, 10 μL of red blood cell homogenate was used in the reaction system. The consumption of H_2_O_2_ was measured at 240 nm for 10 s using a spectrophotometer (Shimadzu TCC 240A; Kyoto, Japan). Blank reactions were composed of the reaction system without H_2_O_2_ and without homogenate. One unit (U) of CAT was defined as the amount of enzyme required to decompose 1 μmol of H_2_O_2_/min.

##### GPx Assay

The enzymatic activity of GPx was determined by monitoring nicotinamide adenine dinucleotide phosphate (NADPH) oxidation at 340 nm for 60 s [30]. Specifically, 30 μL of red blood cell homogenate was used in the reaction system. Blank reactions were composed of the reaction system without H_2_O_2_ and without sample. One unit (U) of GPx was defined as the amount of enzyme required to oxidize 1 nmol of NADPH/min.

##### Glutathione-S-Transferase (GST) Assay

GST activity was determined by monitoring S-2,4-dinitrophenyl-glutathione complex formation at 340 nm for 60 s [30]. Specifically, 30 μL of red blood cell homogenate was used in the reaction system. Blank reactions were carried out without GSH and without homogenate. One unit (U) of GST was defined as the amount of enzyme required to produce 1 nmol of the product/min [31].

##### SOD Assay

The specific activity of the SOD enzyme was determined as described by McCord and Fridovic [32]. The reduction in cytochrome C by xanthine oxidase was monitored at 550 nm for 2 min. The reaction system was prepared with red blood cell homogenate in different volumes (10, 25, 50, 75, or 100 µL) and 10 mmol/L xanthine oxidase in sufficient quantity to generate a cytochrome C reduction rate of 0.025 abs/min. One unit (U) of SOD corresponded to the amount of enzyme required to inhibit the reduction in cytochrome C by 50% (IC50).

##### Oxidative Damage to Lipids

The MDA concentration in the serum was determined by detecting the adduct MDA-2-thiobarbituric acid by fluorescence (emission at 553 nm and excitation at 532 nm), as described by Wasowick et al. [33]. Specifically, 400 µL serum homogenate (325 µL serum/975 µL MilliQ water) was used in the reaction system. A standard curve was constructed using the product of the acid hydrolysis of 1,1,3,3-tetraethoxypropane (0.3125–10 nmol/mL; r2 = 0.999), and the results were expressed as nmol MDA/mL of serum.

##### Oxidative Damage to Proteins

The concentration of carbonylated proteins was determined as described by Mesquita et al. [34]. In this assay, NaOH was added to the plasma following the addition of 2,4-dinitrophenylhydrazine, the maximum absorbance shifted to 450 nm. Specifically, 50 μL of red blood cell homogenate was used in the reaction system. Blank reactions were composed of the reaction system without 2,4-dinitrophenylhydrazine or without homogenate. A molar absorption coefficient of 22,308 M^−1^ cm^−1^ was used to quantify the concentration of protein carbonyl groups, with the results expressed as nmol/mg of total protein in plasma.

### 2.6. Statistical Analysis

Variables are expressed as mean and standard deviation or as absolute and relative frequencies. The Shapiro–Wilk test was performed to evaluate the normality of the numeric variables. Principal component analysis (PCA) was performed to identify the redox response patterns. The Kaiser–Meyer–Olkin coefficient was estimated by measuring the adequacy of PCA, and values between 0.5 and 1 were considered acceptable for this index. Therefore, patterns with eigenvalues > 1.2 defined were extracted from the PCA. The structure of the components was obtained from indicators that presented factor loading > 0.30 [19], with a variable generated in scoring units for each redox response pattern. For each pattern, a categorical variable was created using the median scores of these components. The principal components were divided into two groups (below and above the median) due to the sample size, and therefore Mann–Whitney U test was performed. The Mann–Whitney U test was used to compare the differences between two independent groups.

**Figure 2:**
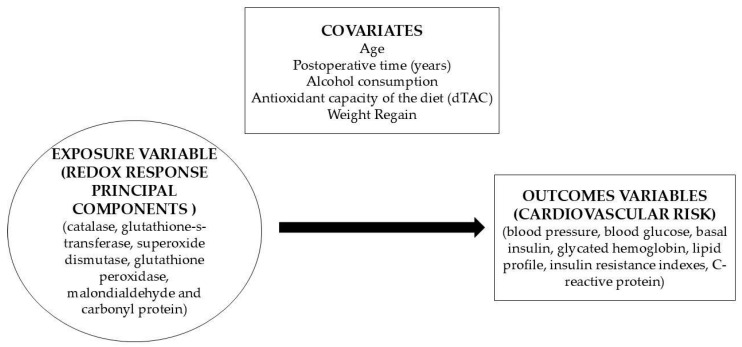
Theoretical model to explore exposure variables, outcome variables and confounding covariates.

To verify the association between the principal component redox response (explanatory variables) and cardiovascular risk outcomes in patients after MBS (dependent variables), crude and adjusted generalized linear models were constructed. Crude models were generated between the exposure and outcome variables and were subsequently analyzed: model 1: adjusted for age, postoperative time, alcohol consumption and dTAC; and model 2: model 1 + weight regain (Figure 2).

Nonstandardized beta coefficients and their respective 95% confidence intervals (95% CI) are presented. All data were analyzed using Stata software (version 16.0, College Station, TX, USA). Statistical significance was set at *p*-value < 0.05.

## 3. Results

The sample mainly comprised women aged approximately 40 years, with an average postoperative time of 4 years (Table 2). Despite the high percentage of excess weight loss and total weight loss in the postoperative period (76.75 and 44.44%, respectively), 59.34% of participants showed significant WR.

Table 3 presents the PCA for redox response biomarkers. Two PC were extracted from the redox response variables during the postoperative period following MBS, together with two patterns representing 49.08% of total variance. PC1 included antioxidant enzymes, whereas PC 2 included carbonylated proteins and MDA.

Body composition and cardiovascular risk variables were compared using two categories: below and above the median of PC1 and PC2 scores, respectively. No differences were found between PC1 or PC2 data and body composition. However, for the cardiovascular risk variables, individuals with enzymatic antioxidant activity (PC1) values above the median had higher CRP levels than those with values below the median (*p* = 0.02). Participants with oxidative damage to proteins and lipids (PC2) values above the sample median had higher diastolic blood pressure (*p* = 0.02), insulin resistance by TyG index (*p* = 0.01), total cholesterol (*p* = 0.008), and serum TGs (*p* = 0.005) than participants with values below the median (Table 4).

Possible associations between the redox variables and cardiovascular risk markers were evaluated using generalized linear regression models (Table 5). No associations were found between PC1 and cardiovascular risk markers according to the crude and adjusted regression model analyses.

For PC2, in the crude model, it was observed that higher scores (above the median) of oxidative damages were associated with higher mean values of systolic blood pressure (β: 7.18, 95% CI: 0.34; 14.03; *p* = 0.04), diastolic blood pressure (β: 6.79, 95% CI: 1.97; 11.61; *p* = 0.006), TyG index (β: 0.13, 95% CI: 0.05; 0.21; *p* = 0.002), total cholesterol (β: 15.17, 95% CI: 3.61; 26.72; *p* = 0.01), serum TGs (β: 25.88, 95% CI: 8.58; 43.18; *p* = 0.003), non-HDL-C (β: 10.91, 95% CI: 0.02; 21.88; *p* = 0.05), and CRP (β: 1.48, 95% CI: 0.01; 2.95; *p* = 0.05). These associations remained significant after adjusting for models 1 and 2, except for excluding systolic blood pressure and CRP levels. Although non-HDL-C cholesterol did not have a significant association in Model 1, there was significance in Model 2 (Table 5).

## 4. Discussion

This study demonstrated a positive association between oxidative damage variables and markers of cardiovascular risk, such as diastolic blood pressure, TyG index, TGs, total cholesterol, and non-HDL-C, in the late postoperative period after MBS. Some of the potential of this work lies in the evaluation of redox response variables using PCA, which consists of a factorial analytical method that optimizes the information observed in a smaller number of variables with minimal loss of observation [19]. In this study, all antioxidant markers were grouped into PC1, whereas markers of oxidative damage were grouped into another component (PC2). It should be noted that the PCs resulted from exploratory statistical groupings, and not from validated biological pathways in this study. The markers CAT, GPx, GST, and SOD, which were grouped into PC1, constitute the primary antioxidant defenses of humans, catalyzing a series of chemical reactions in which the product of one reaction is the substrate for the other, resulting in fewer reactive molecules [35]. Furthermore, PC2 grouped two markers that are products of ROS-catalyzed oxidative damage, such as MDA, one of the end products of polyunsaturated fatty acid peroxidation, and carbonyls, a product of amino acid chain-catalyzed oxidation [33].

Although redox homeostasis is instrumental in the pathophysiology of cardiovascular diseases [36], no association was observed between enzymatic antioxidant activity (PC1) and cardiovascular risk in the late postoperative period after MBS. Similar to our results, Drăgoi et al., while reviewing the function of antioxidants in maintaining redox balance in cardiovascular diseases, also did not observe differences in SOD and CAT activity in heart failure [37]. Recent studies [38,39] have discussed the controversial relationship between antioxidants and cardiovascular diseases. A meta-analysis showed that some compounds promote cardiovascular risk reduction, others have no effects, and some may even increase cardiovascular risk [40]. The authors suggested that antioxidants with strong evidence of reduction in cardiovascular risk are those with pleiotropic properties and that, in addition to antioxidant capacity, they have anti-inflammatory and anti-platelet effects [40]. Furthermore, non-enzymatic antioxidants play an important role and act in conjunction with the enzymatic antioxidant system [41]. Future studies should explore the relationship of antioxidant enzymes and other compounds involved in the control of oxidative stress.

However, a positive association was found between PC2, consisting of MDA and carbonyl proteins, and blood pressure, insulin resistance (TyG index), serum TG, total cholesterol, and non-HDL-C levels. One hypothesis is that the elevated generation of ROS is influencing the increased cardiovascular risk rather than antioxidant enzymatic defenses. Possibly, the products of lipid and protein oxidation reflect the actual action of ROS, rather than merely their production. From this perspective, markers such as MDA and carbonyl proteins indicate oxidative processes that have already occurred, and their presence may trigger a series of metabolic pathways associated with increased insulin resistance [42], hypertension [43], and cardiovascular risk [36]. It is important to highlight that the mechanisms mentioned are possible hypotheses that need to be further investigated in future studies.

Corroborating these data, Zuin et al. [44] showed that individuals with hypertension presented higher serum MDA levels than did normotensive individuals (4.91 and 3.43 nmol/L, respectively), suggesting that MDA may be a biomarker of oxidative stress and endothelial dysfunction. In another recent study, the authors confirmed the potential of MDA as a biomarker for predicting coronary artery disease severity [45]. In addition, a recent cohort study that used gamma-glutamyl transferase activity and uric acid concentration as oxidative stress biomarkers found that these variables mediated 10.53% of association between TyG index, an insulin resistance biomarker, and mortality from cardiovascular disease in adults with metabolic syndrome [46]. Increased oxidative stress, as measured by MDA and protein carbonyl levels, may contribute to increased cardiovascular risk years after MBS.

In this study, a significant association between oxidative damage and CRP levels was observed, but only in the crude model. The loss of association after model adjustment may be related to the nonspecific nature of CRP as a marker of inflammation in cardiovascular risk assessment [47].

A positive association was observed between oxidative damage and concentrations of serum TGs, total cholesterol, and non-HDL-C, possibly explained by several interrelated biological mechanisms. Concomitantly, the increase in insulin resistance and blood pressure may have contributed to an increase in lipolysis and free fatty acids, also promoting changes in the lipid profile [48]. Other authors have reported MDA levels associated with total cholesterol in apparently healthy women [49]. However, this hypothesis and the interrelationship between these cardiovascular risk markers also need to be confirmed in longitudinal studies.

Collectively, investigating the redox response following MBS provides subclinical screening for signs of weight regain before major changes appear in conventional clinical markers of cardiovascular risk. Long-term clinical follow-up is essential for adjusting dietary patterns, using supplements, encouraging physical activity, and controlling weight regain. These factors can prevent the worsening of the long-term redox response after MBS [50].

The results from this study should be interpreted while accounting for some limitations. First, as a cross-sectional study, it does not allow for establishing a causal relationship between exposure and outcome variables. Second, the predominantly female sample (92%) limits the generalizability of the results across genders. Future longitudinal studies with a representative sample are needed to address this. Third, we did not perform direct measurements of micronutrient status such as zinc, copper, selenium, and vitamins A, C, and E (markers of nonenzymatic antioxidant response). Fourth, we also did not investigate other inflammatory adipokines, such as IL-6, MCP-1, and NF-kb. Although we have investigated the association of the inflammatory marker CRP with the redox response, we cannot rule out the possible interference of other uninvestigated inflammatory factors. Fifth, although the KMO index obtained in this study, a value considered borderline indicates a moderate adequacy of the sample for factor analysis; even though the value is higher than the minimum limit of 0.5, it suggests that the correlations between the variables are not as strong as they would ideally be, which may affect the clarity and reliability of the identified factor structure. Therefore, although the analysis was possible, the results should be interpreted with caution, and future studies with more adequate samples could offer a more robust validation of the conclusions.

In contrast, our study has several strengths such as the measurement of multiple markers of redox response according to rigorous standardized protocols. It is one of the few studies that have evaluated the association between redox response and cardiovascular risk years after MBS.

## 5. Conclusions

Oxidative damage markers (MDA and protein carbonyl) are positively associated with diastolic blood pressure, TyG index, TG, total cholesterol, and non-HDL-C levels following MBS. However, primary enzymatic antioxidant defenses are not associated with these cardiovascular risk markers. The causal mechanisms for the worsening redox response years following MBS are still unclear, and further studies are required to confirm and elucidate these findings.

## Figures and Tables

**Table 1:** Summary table listing biomarkers, analytical methods, and measurement units used in this study.

Biomarkers	Analytical Methods	Measurement Units
**Antioxidant response**		
Glutathione-S-transferase (GST)	Spectrophotometry	U/mg protein
Superoxide dismutase (SOD)	Spectrophotometry	U/mg protein
Catalase (CAT)	Spectrophotometry	U/mg protein
GPx (Glutathione Peroxidase)	Spectrophotometry	U/mg protein
**Oxidative Damage**		
Carbonyl protein	Spectrophotometry	nmol/mg protein
Malondialdehyde (MDA)	Fluorimetry	nmol/mL

**Table 2:** Demographic, clinical characteristics and redox response of a sample after MBS (n = 91).

Variables	All (*n* = 91)
**Demographic**	
Female (n/%)	84 (92.31)
Age (years)	39.82 ± 7.87
Educational level (years)	13 ± 2.3
**Clinical characteristics**	
Postoperative time (years)	3.95 ± 1.48
Roux-en-Y Gastric Bypass (n/%)	86 (94.51)
Sleeve gastrectomy (n/%)	5 (5.49)
Preoperative body mass index (kg/m^2^)	42.09 ± 5.64
Current body mass index (kg/m^2^)	29.53 ± 5.01
Excess Weight Loss (%)	76.75 ± 25.07
Total Weight Loss (%)	44.44 ± 18.81
Weight regain (%)	54 (59.34)
Self-reported postoperative comorbidities ^a^ (n/%)	16 (17.58)
Self-reported sedentary lifestyle (n/%)	80 (87.91)
Smoking (n/%)	5 (5.49)
High risk of alcohol use disorder ^b^ (n/%)	23 (25.27)
Supplements—Multivitamins (n/%)	65 (71.43)
Supplements—Omega-3 (n/%)	16 (17.58)
Supplements—Iron (n/%)	28 (30.77)
dTAC ^c^ (mmol Fe/1000 kcal)	5.30 ± 3.36
**Redox response**	
**Antioxidant response**	
GST ^d^ (U/mg protein)	3.47 ± 1.53
SOD ^e^ (U/mg protein)	8.48 ± 3.43
CAT ^f^ (U/mg protein)	112.14 ± 20.45
GPx ^g^ (U/mg protein)	31.99 ± 11.86
**Oxidative Damage**	
Carbonyl (nmol/mg protein)	1.26 ± 0.29
MDA ^h^ (nmol/mL)	10.85 ± 2.15

Values presented as mean ± standard deviation or n (%). MBS = Metabolic and bariatric surgery. ^a^ Systemic arterial hypertension, steatosis, hypothyroidism, and scleroderma. ^b^ Measured by Alcohol Use Disorders Identification Test (AUDIT). ^c^ dTAC= Antioxidant capacity of the diet. ^d^ GST = Glutathione-S-transferase. ^e^ SOD = Superoxide dismutase. ^f^ CAT = catalase. ^g^ GPx = Glutathione Peroxidase. ^h^ MDA = Malondialdehyde.

**Table 3:** The main principal components extracted from redox response data set by percentage of explained variance.

Latent/Observed Variables	PC1	PC2
**Redox response**		
GST (U/mg protein)	0.4277	
SOD (U/mg protein)	0.4670	
CAT (U/mg protein)	0.5758	
GPx (U/mg protein)	0.5031	
Carbonyl (nmol/mg protein)		0.3314
MDA (nmol/mL)		0.7757
**Eigenvalue**	1.74	1.20
**Variance explained (%)**	29.08	20.00
**Cumulative explained variance (%)**	29.08	49.08

KMO = 0.584; Note: KMO = Kaiser–Meyer–Olkin. PC = principal component. GST = Glutathione-S-transferase. SOD = Superoxide dismutase. CAT = catalase. GPx = Glutathione Peroxidase. MDA = Malondialdehyde.

**Table 4:** Body composition and cardiovascular risk markers in the total sample and stratified by principal component after MBS.

Variables	Total Sample	PC1 Score Below Median	PC1 Score Above Median	*p*-Value *	PC2 Score Below Median	PC2 Score Above Median	*p*-Value *
**Body Composition**							
Fat-free mass (kg)	49.78 ± 8.22	49.52 ± 8.72	50.05 ± 7.76	0.47	50.16 ± 8.54	49.39 ± 7.95	0.58
Skeletal muscle mass (kg)	27.22 ± 4.90	27.04 ± 5.17	27.41 ± 4.65	0.43	27.36 ± 5.15	27.07 ± 4.68	0.82
Fat mass (kg)	30.47 ± 11.57	30.21 ± 11.44	30.74 ± 11.8	0.81	31.01 ± 13.03	29.93 ± 9,98	0.99
Body fat (%)	36.93 ± 7.80	36.95 ± 7.58	36.91 ± 8.10	0.77	36.89 ± 8.41	36.97 ± 7.22	0.90
VAT (cm^2^) ^a^	118.83 ± 35.94	118.42 ± 36.30	119.23 ± 35.98	0.81	117.92 ± 36.92	119.73 ± 35.33	0.82
**Cardiovascular risk**							
SBP (mmHg) ^b^	113.46 ± 16.67	115.81 ± 19.56	111.11 ± 12.98	0.30	109.95 ± 12.86	117.14 ± 19.38	0.09
DBP (mmHg) ^c^	75.82 ± 11.95	78.18 ± 12.61	73.45 ± 10.89	0.06	72.50 ± 9.19	79.29 ± 13.54	**0.02**
Blood glucose (mg/dL)	80.16 ± 6.79	81.17 ± 5.46	79.13 ± 7.85	0.09	80.30 ± 5.94	80.02 ± 7.62	0.73
Basal insulin (µU/L)	6.42 ± 4.91	6.31 ± 3.96	6.54 ± 5.76	0.74	6.26 ± 4.15	6.59 ± 5.62	0.85
HbA1c (%) ^d^	5.40 ± 0.34	5.39 ± 0.32	5.41 ± 0.36	0.87	5.40 ± 0.35	5.40 ± 0.33	0.72
HOMA-IR ^e^	1.29 ± 1.03	1.27 ± 0.87	1.30 ± 1.18	0.57	1.24 ± 0.89	1.33 ± 1.17	0.94
TyG index ^f^	4.30 ± 0.21	4.31 ± 0.19	4.30 ± 0.23	0.43	4.24 ± 0.16	4.38 ± 0.24	**0.01**
Total Cholesterol (mg/dL)	158.98 ± 28.98	159.30 ± 28.24	158.64 ± 30.03	0.68	151.48 ± 27.40	166.64 ± 28.83	**0.008**
TG (mg/dL) ^g^	75.32 ± 43.84	72.89 ± 28.93	77.80 ± 55.34	0.85	62.52 ± 17.33	88.40 ± 57.25	**0.005**
LDL-C (mg/dL) ^h^	89.13 ± 25.91	90.41 ± 25.40	87.82 ± 26.6	0.58	86.02 ± 25.89	92.31 ± 25.83	0.24
HDL-C (mg/dL) ^i^	56.16 ± 14.48	55.52 ± 12.87	56.82 ± 16.08	0.92	53.72 ± 12.97	58.67 ± 15.62	0.13
Non-HDL-C (mg/dL) ^j^	103.15 ± 27.02	104.39 ± 26.79	101.89 ± 27.49	0.51	97.74 ± 25.72	108.69 ± 27.47	**0.05**
CRP (mg/L) ^k^	1.51 ± 3.64	1.45 ± 4.41	1.58 ± 2.69	**0.02**	0.78 ± 1.13	2.26 ± 4.97	0.20

Values presented as mean ± standard deviation. * *p*-values were derived from the Mann–Whitney U test. Significant values were presented in bold. MBS = Metabolic and bariatric surgery. PC = Principal component. PC1 = characterized by the markers glutathione-S-transferase, superoxide dismutase, catalase and glutathione peroxidase. PC2: characterized by carbonyl proteins and malondialdehyde. ^a^ VAT = Visceral adipose tissue. ^b^ SBP = Systolic blood pressure. ^c^ DBP = Diastolic blood pressure. ^d^ HbA1c = Hemoglobin A1c. ^e^ HOMA-IR = Homeostatic Model Assessment for Insulin Resistance. ^f^ TyG = Triglyceride-glucose index. ^g^ TG = Triglyceride. ^h^ LDL-C = Low-density lipoprotein cholesterol. ^i^ HDL-C = High-density lipoprotein cholesterol. ^j^ Non-HDL-C = Non-high-density lipoprotein cholesterol. ^k^ CRP = C-reactive protein.

**Table 5:** Generalized linear regression models associations between redox response principal components (explanatory variables) and cardiovascular risk outcomes after MBS (dependent variables).

Variables	PC1 ^a^			PC2 ^b^		
	Crude Model ^c^	Model 1 ^d^	Model 2 ^e^	Crude Model ^c^	Model 1 ^d^	Model 2 ^e^
	Coefficient β (95% CI)			Coefficient β (95% CI)		
<median scores	(ref.)	(ref.)	(ref.)	(ref.)	(ref.)	(ref.)
SBP (mmHg) ^f^	−4.70 (−11.64; 2.23)	−3.96 (−10.69; 2.77)	−4.08 (−10.80; 2.63)	7.18 (0.34; 14.03) *	6.19 (−0.53; 12.92)	5.98 (−0.76; 12.71)
DPB (mmHg) ^g^	−4.73 (−9.65; 0.20)	−4.18 (−9.02; 0.66)	−4.29 (−9.11; 0.52)	6.79 (1.97; 11.61) *	6.48 (1.71; 11.26) *	6.30 (1.53; 11.07) *
Blood glucose (mg/dL)	−2.04 (−4.81; 0.73)	−1.92 (−4.70; 0.86)	−1.91 (−4.66; 0.84)	−0.28 (−3.08; 2.52)	−0.30 (−3.14; 2.54)	−0.09 (−2.91; 2.73)
Basal insulin (µU/L)	0.23 (−1.80; 2.25)	−0.17 (−2.13; 1.78)	−0.18 (−2.14; 1.79)	0.33 (−1.69; 2.36)	0.57 (−1.42; 2.53)	0.50 (−1.49; 2.50)
HbA1c (%) ^h^	0.01 (−0.12; 0.16)	0.004 (−0.12; 0.13)	0.004 (−0.13; 0.13)	0.002 (−0.14; 0.14)	0.04 (−0.09; 0.17)	0.04 (−0.09; 0.17)
HOMA-IR ^i^	0.03 (−0.40; 0.45)	−0.05 (−0.47; 0.36)	−0.05 (−0.47; 0.36)	0.08 (−0.34; 0.51)	0.13 (−0.28; 0.55)	0.12 (−0.30; 0.55)
TyG ^j^	−0.007 (−0.09; 0.08)	−0.008 (−0.10; 0.08)	−0.008 (−0.10; 0.08)	0.13 (0.05; 0.21) *	0.13 (0.05; 0.22) *	0.13 (0.05; 0.22) *
Total Cholesterol (mg/dL)	−0.66 (−12.63; 11.31)	0.71 (−10.28; 11.69)	0.75 (−10.12; 11.62)	15.17 (3.61; 26.72) *	13.79 (3.07; 24.51) *	14.69 (4.11; 25.28) *
TG (mg/dL) ^k^	4.91 (−13.18; 23.00)	4.07 (−14.04; 22.19)	4.08 (−14.14; 22.30)	25.88 (8.58; 43.18) *	26.58 (9.13; 44.04) *	26.95 (9.34; 44.55) *
LDL-C (mg/dL) ^l^	−2.59 (−13.28; 8.10)	−2.14 (−12.31; 8.02)	−2.12 (−12.26; 8.03)	6.29 (−4.34; 16.92)	4.90 (−5.35; 15.14)	5.46 (−4.78; 15.71)
HDL-C (mg/dL) ^m^	1.30 (−4.68; 7.28)	2.13 (−3.50; 7.76)	2.14 (−3.52; 7.80)	4.95 (−0.94; 10.84)	5.23 (−0.37; 10.84)	5.39 (−0.26; 11.04)
Non-HDL-C (mg/dL) ^n^	−2.50 (−13.66; 8.65)	−2.08 (−12.51; 8.34)	−2.05 (−12.40; 8.31)	10.91 (0.02; 21.88) *	9.56 (−0.79; 19.92)	10.30 (0.01; 20.59) *
CRP (mg/L) ^o^	0.12 (−1.38; 1.63)	−0.002 (−1.52; 1.52)	−0.007 (−1.52; 1.51)	1.48 (0.01; 2.95) *	1.39(−0.12; 2.90)	1.31 (−0.20; 2.82)

* *p*-Value < 0.05; ^a^ PC1: characterized by the markers glutathione-S-transferase, superoxide dismutase, catalase and glutathione peroxidase. Category 1 (below the median—reference); Category 2: above the median. Scores above the median represent a greater quantity of glutathione-S-transferase, superoxide dismutase, catalase and glutathione peroxidase markers, therefore, a better capacity antioxidant.; ^b^ PC2: characterized by carbonyl proteins and malondialdehyde. Category 1 (below the median—reference); Category 2: above the median. Scores above the median represent a greater quantity of carbonyl protein and MDA markers, therefore, representing worse oxidative damage. ^c^ Crude model was unadjusted. ^d^ Model 1 was adjusted for age, postoperative time, alcohol consumption and antioxidant capacity of the diet. ^e^ Model 2 was adjusted for age, postoperative time, alcohol consumption, antioxidant capacity of the diet and weight regain. Note: PC = Principal component. CI = Confidence interval. ^f^ SBP = Systolic blood pressure. ^g^ DBP = Diastolic blood pressure. ^h^ HbA1c = Hemoglobin A1c. ^i^ HOMA-IR = Homeostatic Model Assessment for Insulin Resistance. ^j^ TyG = Triglyceride-glucose index. ^k^ TG = Triglyceride. ^l^ LDL-C = Low-density lipoprotein cholesterol. ^m^ HDL-C = High-density lipoprotein cholesterol. ^n^ Non-HDL-C = Non-high-density lipoprotein cholesterol. ^o^ CRP = C-reactive protein.

## Data Availability

The data used in this study are available from the corresponding. Author upon reasonable request. The data are not publicly available due to privacy reasons.

## Abbreviations

The following abbreviations are used in this manuscript:

MBS	Metabolic and bariatric surgery
ROS	Reactive oxygen species
TBARSs	Thiobarbituric acid reactive substances
WR	Weight regain
BMI	Body mass index
dTAC	Total antioxidant capacity of the diet
TyG	Triglyceride/glucose index
TG	Triglyceride
HDL-C	High-density lipoprotein cholesterol
LDL-C	Low-density lipoprotein cholesterol
CRP	C-reactive protein
Non-HDL-C	Non-high-density lipoprotein cholesterol
PCA	Principal component analysis
PC	Principal component
SOD	Superoxide dismutase
GPx	Glutathione peroxidase
GST	Glutathione-S-transferase
CAT	Catalase
MDA	Malondialdehyde

## References

[1] Yu J., Qiu J., Zhang Z., Cui X., Guo W., Sheng M., Gao M., Wang D., Xu L., Ma X. (2023). Redox Biology in Adipose Tissue Physiology and Obesity. Adv. Biol..

[2] Masenga S.K., Kabwe L.S., Chakulya M., Kirabo A. (2023). Mechanisms of Oxidative Stress in Metabolic Syndrome. Int. J. Mol. Sci..

[3] Soldo A.M., Soldo I., Karačić A., Konjevod M., Perkovic M.N., Glavan T.M., Luksic M., Žarković N., Jaganjac M. (2022). Lipid Peroxidation in Obesity: Can Bariatric Surgery Help?. Antioxidants.

[4] Pradel-Mora J.J., Marín G., Castillo-Rangel C., Hernández-Contreras K.A., Vichi-Ramírez M.M., Zarate-Calderon C., Motta F.S.H. (2024). Oxidative Stress in Postbariatric Patients: A Systematic Literature Review Exploring the Long-term Effects of Bariatric Surgery. Plast. Reconstr. Surg. Glob. Open.

[5] Hua Y., Lou Y.X., Li C., Sun J.Y., Sun W., Kong X.Q. (2022). Clinical outcomes of bariatric surgery—Updated evidence. Obes. Res. Clin. Pract..

[6] Perdomo C.M., Cohen R.V., Sumithran P., Clément K., Frühbeck G. (2023). Contemporary medical, device, and surgical therapies for obesity in adults. Lancet.

[7] Pinto S.L., Juvanhol L.L., de Oliveira L.L., Clemente R.C., Bressan J. (2019). Changes in oxidative stress markers and cardiometabolic risk factors among Roux-en-Y gastric bypass patients after 3- and 12-months postsurgery follow-up. Surg. Obes. Relat. Dis..

[8] Dadalt C., Fagundes R.L.M., Moreira E.A.M., Wilhelm-Filho D., De Freitas M.B., Jordão Júnior A.A., Biscaro F., Pedrosa R.C., Vannucchi H. (2013). Oxidative stress markers in adults 2 years after Roux-en-Y gastric bypass. Eur. J. Gastroenterol. Hepatol..

[9] Tozzo C., Moreira E.A.M., de Freitas M.B., da Silva A.F., Portari G.V., Wilhelm Filho D. (2020). Effect of RYGB on Oxidative Stress in Adults: A 6-Year Follow-up Study. Obes. Surg..

[10] Cui B., Wang G., Li P., Li W., Song Z., Sun X., Zhu L., Zhu S. (2023). Disease-specific mortality and major adverse cardiovascular events after bariatric surgery: A meta-analysis of age, sex, and BMI-matched cohort studies. Int. J. Surg..

[11] El Ansari W., Elhag W. (2021). Weight Regain and Insufficient Weight Loss After Bariatric Surgery: Definitions, Prevalence, Mechanisms, Predictors, Prevention and Management Strategies, and Knowledge Gaps—A Scoping Review. Obes. Surg..

[12] Athanasiadis D.I., Martin A., Kapsampelis P., Monfared S., Stefanidis D. (2021). Factors associated with weight regain post-bariatric surgery: A systematic review. Surg. Endosc..

[13] Ben-Porat T., Mashin L., Kaluti D., Goldenshluger A., Shufanieh J., Khalaileh A., Gazala M.A., Mintz Y., Brodie R., Sakran N. (2021). Weight Loss Outcomes and Lifestyle Patterns Following Sleeve Gastrectomy: An 8-Year Retrospective Study of 212 Patients. Obes. Surg..

[14] Courcoulas A.P., Daigle C.R., Arterburn D.E. (2023). Long term outcomes of metabolic/bariatric surgery in adults. BMJ.

[15] King W.C., Hinerman A.S., Belle S.H., Wahed A.S., Courcoulas A.P. (2018). Comparison of the Performance of Common Measures of Weight Regain after Bariatric Surgery for Association with Clinical Outcomes. JAMA—J. Am. Med. Assoc..

[16] Jirapinyo P., Dayyeh B.K.A., Thompson C.C. (2017). Weight regain after Roux-en-Y gastric bypass has a large negative impact on the Bariatric Quality of Life Index. BMJ Open Gastroenterol..

[17] Courcoulas A.P., King W.C., Belle S.H., Berk P., Flum D.R., Garcia L., Gourash W., Horlick M., Mitchell J.E., Pomp A. (2018). Seven-year weight trajectories and health outcomes in the Longitudinal Assessment of Bariatric Surgery (LABS) study. JAMA Surg..

[18] King W.C., Hinerman A.S., Courcoulas A.P. (2020). Weight regain after bariatric surgery: A systematic literature review and comparison across studies using a large reference sample. Surg. Obes. Relat. Dis..

[19] Hair J.F., Black B., Babin B., Anderson R.E., Tatham R.L. (2009). Multivariete Data Analysis.

[20] Lima C.T., Freire A.C.C., Silva A.P.B., Teixeira R.M., Farrell M., Prince M. (2005). Concurrent and construct validity of the audit in an urban Brazillian sample. Alcohol. Alcohol..

[21] WHO (2000). Obesity: Preventing and managing the global epidemic. Report of a WHO Consultation.

[22] Muntner P., Shimbo D., Carey R.M., Charleston J.B., Gaillard T., Misra S., Myers M.G., Ogedegbe G., Schwartz J.E., Townsend R.R. (2019). Measurement of blood pressure in humans: A scientific statement from the American Heart Association. Hypertension.

[23] Conway J.M., Ingwersen L.A., Vinyard B.T., Moshfegh A.J. (2003). Effectiveness of the US Department of Agriculture 5-step multiple-pass method in assessing food intake in obese and nonobese women. Am. J. Clin. Nutr..

[24] Barufaldi L.A., Abreu G.D.A., Veiga GVDa Sichieri R., Kuschnir M.C.C., Cunha D.B., Pereira R.A., Bloch K.V. (2016). Programa para registro de recordatório alimentar de 24 horas: Aplicação no Estudo de Riscos Cardiovasculares em Adolescentes. Rev. Bras. De Epidemiol..

[25] Harttig U., Haubrock J., Knüppel S., Boeing H. (2011). The MSM program: Web-based statistics package for estimating usual dietary intake using the multiple source method. Eur. J. Clin. Nutr..

[26] Carlsen M.H., Halvorsen B.L., Holte K., Bøhn S.K., Dragland S., Sampson L., Willey C., Senoo H., Umezono Y., Sanada C. (2010). The total antioxidant content of more than 3100 foods, beverages, spices, herbs and supplements used worldwide. Nutr. J..

[27] Matthews D.R., Hosker J.P., Rudenski A.S., Naylor B.A., Treacher D.F., Turner R.C. (1985). Homeostasis model assessment: Insulin resistance and β-cell function from fasting plasma glucose and insulin concentrations in man. Diabetologia.

[28] Guerrero-Romero F., Simental-Mendía L.E., González-Ortiz M., Martínez-Abundis E., Ramos-Zavala M.G., Hernández-González S.O., Jacques-Camarena O., Rodríguez-Morán M. (2010). The product of triglycerides and glucose, a simple measure of insulin sensitivity. Comparison with the euglycemic-hyperinsulinemic clamp. J. Clin. Endocrinol. Metab..

[29] Hartree E.F. (1972). Determination of Protein: A Modification of the Lowry Method That Gives a Linear Photometric Response. Anal. Biochem..

[30] Joanisse D.R., Storey K.B. (1996). Oxidative damage and antioxidants in Rana sylvatica, the freeze-tolerant wood frog. Am. J. Physiol. Regul. Integr. Comp. Physiol..

[31] Habig W.H., Jakoby W.B. (1981). Assays for Differentiation of Glutathione S-Transferases. Methods Enzymol..

[32] McCord J.M. (1999). Analysis of Superoxide Dismutase Activity. Curr. Protoc. Toxicol..

[33] Wasowicz W., Neve J., Peretz A. (1993). Optimized steps in fluorometric determination of thiobarbituric acid-reactive substances in serum: Importance of extraction pH and influence of sample preservation and storage. Clin. Chem..

[34] Mesquita C.S., Oliveira R., Bento F., Geraldo D., Rodrigues J.V., Marcos J.C. (2014). Simplified 2,4-dinitrophenylhydrazine spectrophotometric assay for quantification of carbonyls in oxidized proteins. Anal. Biochem..

[35] Matés J.M., Pérez-Gómez C., De Castro I.N. (1999). Antioxidant enzymes and human diseases. Clin. Biochem..

[36] Jin S., Kang P.M. (2024). A Systematic Review on Advances in Management of Oxidative Stress-Associated Cardiovascular Diseases. Antioxidants.

[37] Drăgoi C.M., Diaconu C.C., Nicolae A.C., Dumitrescu I.B. (2024). Redox Homeostasis and Molecular Biomarkers in Precision Therapy for Cardiovascular Diseases. Antioxidants.

[38] Wan S., Wu W., Zhang Y., He J., Wang X., An P., Luo J., Zhu Y., Luo Y. (2024). Lipid Supplement on Cardiovascular Risk Factors: A Systematic Review and Meta-Analysis. Nutrients.

[39] Gormaz J.G., Carrasco R. (2022). Antioxidant Supplementation in Cardiovascular Prevention: New Challenges in the Face of New Evidence. J. Am. Coll. Cardiol..

[40] An P., Wan S., Luo Y., Luo J., Zhang X., Zhou S., Xu T., He J., Mechanick J.I., Wu W.C. (2022). Micronutrient Supplementation to Reduce Cardiovascular Risk. J. Am. Coll. Cardiol..

[41] Jena A.B., Samal R.R., Bhol N.K., Duttaroy A.K. (2023). Cellular Red-Ox system in health and disease: The latest update. Biomed. Pharmacother..

[42] Boura-Halfon S., Zick Y. (2009). Phosphorylation of IRS proteins, insulin action, and insulin resistance. Am. J. Physiol. Endocrinol. Metab..

[43] Griendling K.K., Camargo L.L., Rios F.J., Alves-Lopes R., Montezano A.C., Touyz R.M. (2021). Oxidative Stress and Hypertension. Circ. Res..

[44] Zuin M., Capatti E., Borghi C., Zuliani G. (2022). Serum Malondialdehyde Levels in Hypertensive Patients: A Non-invasive Marker of Oxidative Stress. A Systematic Review and Meta-analysis. High. Blood Press. Cardiovasc. Prev..

[45] Hadi N., Zaidi I.A., Kamal Z., Khan R.U. (2024). Estimation of Serum Malondialdehyde (a Marker of Oxidative Stress) as a Predictive Biomarker for the Severity of Coronary Artery Disease (CAD) and Cardiovascular Outcomes. Cureus.

[46] Yang M., Shangguan Q., Xie G., Sheng G., Yang J. (2024). Oxidative stress mediates the association between triglyceride-glucose index and risk of cardiovascular and all-cause mortality in metabolic syndrome: Evidence from a prospective cohort study. Front. Endocrinol..

[47] Pepys M.B., Hirschfield G.M. (2003). C-reactive protein: A critical update. J. Clin. Investig..

[48] Kosmas C.E., Bousvarou M.D., Kostara C.E., Papakonstantinou E.J., Salamou E., Guzman E. (2023). Insulin resistance and cardiovascular disease. J. Int. Med. Res..

[49] Černiauskas L., Mažeikienė A., Mazgelytė E., Petrylaitė E., Linkevičiūtė-Dumčė A., Burokienė N., Karčiauskaitė D. (2023). Malondialdehyde, Antioxidant Defense System Components and Their Relationship with Anthropometric Measures and Lipid Metabolism Biomarkers in Apparently Healthy Women. Biomedicines.

[50] Sharifi-Rad M., Anil Kumar N.V., Zucca P., Varoni E.M., Dini L., Panzarini E., Rajkovic J., Fokou P.V.T., Azzini E., Peluso I. (2020). Lifestyle, Oxidative Stress, and Antioxidants: Back and Forth in the Pathophysiology of Chronic Diseases. Front. Physiol..

